# Artificial Intelligence for Improving the Management of People With Epilepsy in Low‐and‐Middle Income Countries

**DOI:** 10.1111/ene.70740

**Published:** 2026-09-08

**Authors:** Philippe Ryvlin, Sándor Beniczky, Harald Aurlien, Adriano Bernini, Gabriel Davis Jones, Symon M. Kariuki, Antoine Spahr, Jesper Tveit, Arjune Sen

**Affiliations:** ^1^ Department of Clinical Neurosciences – Member of the European Reference Network EpiCARE Centre Hospitalier Universitaire Vaudois et Université de Lausanne Lausanne Switzerland; ^2^ Department of Clinical Neurophysiology Danish Epilepsy Center, Filadelfia – Member of the European Reference Network EpiCARE, Copenhagen University Hospital, Rigshospitalet and University of Copenhagen Copenhagen Denmark; ^3^ Department of Clinical Neurophysiology Haukeland University Hospital Bergen Norway; ^4^ Natus Medical Bergen Norway; ^5^ Centre for Global Epilepsy, Wolfson College, and Nuffield Department of Clinical Neurosciences University of Oxford Oxford UK; ^6^ Early Childhood Development Unit African Population and Health Research Center Nairobi Kenya

**Keywords:** apps, digital technology, EEG, Mobile health, seizure detection

## Abstract

**Background:**

About 80% of people with epilepsy live in low‐and‐middle‐income countries (LMICs) where the treatment gap is high. Limited access to neurologists, diagnostic tools, and antiseizure medications, combined with persistent stigma, contribute to poor outcomes, including premature mortality. Artificial intelligence (AI) offers potential to address these gaps through scalable, low‐cost solutions for diagnosis, investigation, management, and monitoring.

**Methods:**

This review examines three recent and complementary AI applications in epilepsy care for LMICs: a smartphone‐based diagnostic tool for convulsive epilepsy developed using population‐based data from five sub‐Saharan African countries; an automated EEG interpretation system based on a deep learning model (SCORE‐AI) validated across multicenter datasets; and a wearable‐based deep learning model for detecting generalized convulsive seizures using low‐cost smartwatches.

**Results:**

The smartphone diagnostic tool achieved area under the curve (AUC) 0.92–0.95 with sensitivity 85.0%–97.5% for identifying epilepsy with convulsive seizures using eight binary clinical features. SCORE‐AI demonstrated expert‐level performance (AUC 0.89–0.96, accuracy 85%–92%) for automated EEG classification across multiple validation datasets. The wearable seizure detection algorithm achieved 96% sensitivity with approximately one false alarm per 8 days. All three solutions were designed for deployment on widely accessible platforms.

**Conclusions:**

AI‐driven approaches demonstrate feasibility for addressing diagnostic and monitoring gaps in resource‐limited settings. However, implementation faces substantial challenges including infrastructure constraints, limited digital literacy, ethical considerations, and sociocultural factors. Successful deployment requires validation with large locally relevant datasets, context‐adapted solutions, task‐sharing strategies, implementation research, appropriate regulatory frameworks/certification, and community engagement to reduce the global epilepsy care gap.

## 1 Introduction

Epilepsy is one of the most common neurological disorders worldwide, contributing substantially to global disability and premature mortality [1, 2]. Strikingly, nearly 80% of people with epilepsy (PWE) reside in low‐ and middle‐income countries (LMICs), where the burden of disease is disproportionately elevated and outcomes remain markedly worse than in high‐income settings [2, 3]. Premature mortality rates are increased in PWE from LMICs, with a majority of them ascribed to the epilepsy treatment gap. This higher mortality reflects a complex interplay between limited access to care, delayed and erroneous diagnosis, and suboptimal treatment [4]. In addition to its health impact, epilepsy imposes a significant socioeconomic burden, accounting for millions of disability‐adjusted life years (DALYs) annually and substantial direct and indirect costs in LMICs [5].

A central challenge in LMICs is the epilepsy treatment gap (ETG), defined as the proportion of individuals with active epilepsy who do not receive appropriate treatment [1, 6]. This gap ranges from approximately 50% to nearly 100% in resource‐limited regions, with a pooled estimate of 71% across LMICs [6, 7]. A systematic review by the ILAE Epidemiology Commission found treatment gaps ranging from 5.6% in Norway to 100% in parts of Tibet, Togo, and Uganda [6]. The gap is particularly pronounced in rural and underserved populations or areas, which consistently show higher treatment gaps than urban areas [8].

Multiple factors contribute to this ETG. First, there is a critical shortage of trained neurologists and epileptologists, often compounded by unequal geographic distribution of healthcare professionals [9, 10, 11]. In sub‐Saharan Africa, there is an estimated average of only 0.6 neurologists per million people, with 10 African nations having no neurologists at all [10, 11]. Second, access to essential diagnostic aid tools, most notably electroencephalography (EEG) and neuroimaging, is severely limited [12, 13, 14]. A survey across 37 countries found that neurodiagnostic tests are least affordable in the lowest income settings, with only the top 10%–20% of the population in low‐income countries able to afford tests [14]. Even when available, these investigations are frequently inaccessible owing to cost, infrastructure constraints, lack of appropriate expertise for interpretation, or logistical barriers [13, 14]. Consequently, epilepsy diagnosis in LMICs often relies heavily on clinical history alone, increasing the risk of misdiagnosis or delayed recognition, particularly when investigations are needed to rule out other paroxysmal mimics of epileptic seizures [12].

Misdiagnosis represents a major but underappreciated issue. Distinguishing epileptic seizures from non‐epileptic events, such as syncope or functional dissociative seizures (FDS), requires expertise and support‐tools that are frequently lacking in resource‐limited settings [15, 16]. Errors in diagnosis may lead either to inappropriate treatment or to the absence of necessary therapy, both of which carry significant consequences for patients and caregivers [1, 12]. Even when a diagnosis is established, access to essential antiseizure medications remains inconsistent [17, 18]. Drug availability is often unreliable, and affordability remains a major barrier, with treatment costs representing a substantial proportion of household income in some settings [17, 18, 19].

Beyond healthcare system limitations, sociocultural factors further exacerbate the psychosocial burden of epilepsy in LMICs [20, 21, 22]. Persistent stigma, misconceptions about disease causation, and reliance on traditional healing practices frequently delay or prevent engagement with formal healthcare systems [13, 22, 23]. In many communities, epilepsy is still associated with supernatural beliefs, leading to discrimination, social exclusion, and reduced educational and employment opportunities [21, 22, 23]. These factors not only impair quality of life but also contribute to poor adherence to treatment and follow‐up. Importantly, stigma can be internalized, resulting in reluctance to seek care even when services are available [8, 13].

Taken together, these challenges highlight the need for innovative, scalable, and context‐adapted solutions to reduce the epilepsy care gap in LMICs. Recently, artificial intelligence (AI) has emerged as a promising tool with the potential to transform epilepsy care delivery [3, 24, 25, 26, 27]. By leveraging advances in machine learning and digital health technologies, AI‐based systems can support key steps along the epilepsy care pathway, including diagnosis, investigation, management, and long‐term monitoring [3, 24, 25, 26, 27, 28]. Importantly, these tools can be designed to operate with minimal infrastructure, making them particularly suited for deployment in resource‐constrained environments [13, 29].

One of the most compelling advantages of AI and digital technology in LMICs is its capacity to support task‐sharing strategies [12, 13]. By assisting non‐specialist healthcare workers in performing activities traditionally reserved for experts, such as identifying seizure characteristics, interpreting EEG recordings, or detecting seizures, AI can extend the reach of specialized and expertise services beyond tertiary centers [13, 24]. This approach aligns closely with global health strategies, including the WHO Intersectoral Global Action Plan (IGAP) and Mental Health Gap Action Program (mhGAP), aimed at strengthening primary care and decentralizing specialized services [29]. In addition, AI‐driven tools can standardize clinical assessments, reduce interobserver variability, and potentially improve diagnostic accuracy in settings where expertise is scarce [30, 31].

Another key opportunity lies in the integration of AI with widely available technologies such as smartphones and wearable devices [12, 13, 29, 32]. Mobile health platforms can facilitate data collection, guide clinical decision‐making, and enable remote supervision or referral [12, 13, 25]. Similarly, wearable sensors combined with AI algorithms can provide continuous monitoring and automated detection of seizures, offering new avenues for improving patient safety, optimizing therapy, and generating objective clinical data [24, 26]. These approaches are particularly attractive in LMICs, where mobile device penetration is rapidly increasing despite limitations in traditional healthcare infrastructure [12, 13].

While the theoretical potential of AI in epilepsy care is considerable, its real‐world implementation in LMICs remains in its early stages [25, 26, 31]. There is a need to move beyond conceptual frameworks and demonstrate the feasibility, effectiveness, and scalability of specific AI‐driven solutions in diverse settings [26]. In this regard, several recent initiatives provide concrete examples of how AI can be applied to address key gaps in epilepsy care [25].

In this selective expert review, we present three examples that illustrate distinct but complementary applications of AI in epilepsy care. First, we describe a smartphone‐based diagnostic tool designed to improve the identification of generalized and focal‐to‐bilateral tonic–clonic seizures, referred herein as generalized convulsive seizures (GCS) for readability in sub‐Saharan Africa. Second, we examine the use of AI for automated interpretation of EEG recordings, with the potential to mitigate the shortage of EEG expertise. Third, we discuss the development of a wearable‐based AI system for GCS detection, which may enhance patient monitoring and safety. Together, these examples highlight the transformative potential of AI while providing a foundation for discussing its broader implications in LMIC contexts.

## 2 AI‐Assisted Diagnosis of GCS in LMIC

GCS are the most readily recognized seizure type in sub‐Saharan Africa [33], yet their diagnosis often depends on non‐specialist healthcare workers and incomplete clinical histories. This contributes to delayed or missed diagnoses and underscores the need for diagnostic tools that are both accurate and practical in resource‐constrained settings. To address this challenge, a machine learning–based diagnostic tool was developed to identify individuals with GCS using simple, reproducible clinical features that can be reliably assessed by community healthcare workers [34]. The overarching goal was to create a culturally adapted, scalable solution capable of facilitating earlier and more reliable epilepsy diagnosis in LMICs.

Model development relied on the SEEDS (Studies of the Epidemiology of Epilepsy in Demographic Sites) dataset, a large population‐based cohort collected across Kenya, Ghana, South Africa, Tanzania and Uganda [34]. The dataset included 4097 individuals, comprising 1985 participants with confirmed convulsive epilepsy and 2112 matched controls [34]. Diagnosis was established through a standardized three‐stage process, culminating in clinical assessment by trained clinicians, and supported by EEG when available [34].

Approximately 170 candidate variables encompassing clinical history, seizure semiology, and contextual factors were initially considered [34]. Because the intended users were non‐specialist healthcare workers, feature selection prioritized variables that could be collected reliably as simple binary responses in routine community practice [34]. Information gain and correlation‐based feature selection methods ultimately identified a parsimonious set of eight clinical variables for model development [34].

Several machine learning approaches, including logistic regression, support vector machines, naïve Bayes classifiers, and decision trees, were subsequently trained using a 7:3 training‐validation split combined with ten‐fold cross‐validation. Logistic regression was selected as the final model because it offered the best compromise between diagnostic performance, interpretability, and ease of implementation [34].

The model was externally validated using prospectively collected data from Kilifi, Kenya, while a leave‐one‐site‐out (LOSO) strategy was used to assess generalizability across the five participating countries [34]. The final model was embedded into a multilingual Android‐based smartphone application, the Epilepsy Diagnostic Companion, designed for use by community healthcare workers, with outputs providing both probability estimates and binary classification of epilepsy likelihood [34].

The final predictive model demonstrated high diagnostic performance across multiple validation settings. In the internal validation dataset, the model achieved an area under the receiver operating characteristic curve (AUROC) of 0.92 (95% CI 0.91–0.94), with a sensitivity of 85.0% and specificity of 93.7% [34]. Performance remained robust in the external validation cohort, with an AUROC of 0.95 (0.92–0.98), sensitivity of 97.5%, and specificity of 82.4%, indicating strong generalizability [34].

The LOSO analysis further confirmed stable performance across geographical regions, with an AUROC of 0.94 and balanced sensitivity and specificity [34]. Notably, the final model relied on only eight binary clinical features, including key clinical features such as tongue biting, urinary incontinence, loss of awareness, and motor manifestations. These features align with established clinical markers of convulsive seizures, reinforcing the clinical plausibility of the model.

Beyond discrimination, the model also demonstrated good calibration, indicating reliable probability estimates across different levels of predicted risk [34]. Decision curve analysis showed a net clinical benefit compared with a “treat‐all” strategy over a broad range of probability thresholds, supporting its utility in guiding referral and treatment decisions [34].

The implementation of the model into a smartphone‐based application enabled real‐time use in the field. The tool is designed to function on widely available Android devices, with multilingual support and minimal training requirements. By generating structured reports and enabling data storage, the application also offers potential for epidemiological surveillance and longitudinal data collection in LMIC settings.

This work provides a compelling proof of concept for AI‐assisted diagnosis of epilepsy in LMICs, demonstrating that robust predictive models can be developed using locally derived data and implemented in accessible digital tools. Subsequent work has shown the importance of iterative model improvement and the need to validate the approach in local contexts prior to deployment in new populations [35]. It is also vital to extend applicability beyond GCS, noting that diagnosing nonconvulsive seizures can be much more nuanced. Successful implementation will also require integration into existing healthcare systems together with prospective evaluation of clinical impact, cost‐effectiveness, and long‐term performance. Continuous refinement using locally generated data will be essential to maintain diagnostic accuracy, cultural relevance, and scalability across diverse LMIC contexts.

## 3 Automated Interpretation of EEG Using Artificial Intelligence

Access to expert EEG interpretation remains a major limitation in many parts of the world, particularly in LMICs, where the shortage of trained electroencephalographers contributes to delayed diagnosis and treatment. Recent advances in artificial intelligence have made it possible to automate clinically meaningful interpretation of routine EEG recordings, moving beyond the simple detection of abnormalities toward classifications that directly support clinical decision‐making. In this context, the deep learning model SCORE‐AI (Standardized Computer‐based Organized Reporting of EEG–Artificial Intelligence) was developed to classify EEGs as normal or abnormal and to further categorize abnormal recordings according to the presence of epileptiform or non‐epileptiform abnormalities and their distribution (generalized or focal) [34, 36]. The overarching aim was to achieve diagnostic performance comparable to that of human experts while providing a scalable solution to improve access to EEG interpretation worldwide.

SCORE‐AI was trained using a multicenter dataset comprising 30,493 routine EEG recordings annotated by 17 expert electroencephalographers [36]. All recordings were labeled according to the standardized SCORE terminology, providing consistent annotations and clinically relevant output categories. The model combined multichannel EEG signals with basic demographic information (age and sex) to generate five diagnostic outputs: normal or abnormal EEG, and for abnormal recordings, classification as epileptiform‐focal, epileptiform‐generalized, nonepileptiform‐focal, or nonepileptiform‐diffuse. Training was performed using supervised learning with a predefined architecture that remained fixed throughout subsequent validation.

The clinical performance of SCORE‐AI was assessed across several independent validation datasets [36, 37]. Agreement with expert consensus was first evaluated using a multicenter dataset of 100 EEGs independently reviewed by 11 experienced electroencephalographers. A second validation on a large single‐center cohort of 9785 routine EEGs assessed performance under real‐world clinical conditions, while a third benchmarking dataset enabled comparison with previously published AI‐based approaches [36]. To further examine generalizability, the model was evaluated in an external cohort of 104 patients from a geographically distinct population, recorded with different EEG equipment and linked to an independent reference standard derived from electronic health records and longitudinal clinical follow‐up [37]. Throughout all validation studies, the model remained frozen, and diagnostic performance was assessed without any post hoc optimization.

Across these diverse datasets, SCORE‐AI consistently achieved diagnostic performance comparable to that of experienced EEG readers [36]. For the classification of EEG abnormalities, the AUROC ranged from 0.89 to 0.96, with overall diagnostic accuracy between 85% and 92%, depending on the classification task. In the multicenter validation study, agreement between SCORE‐AI and the expert consensus closely matched the inter‐rater agreement observed among the human experts themselves. Concordance was particularly high for normal EEGs and generalized epileptiform abnormalities, whereas agreement for more heterogeneous patterns, including focal and nonepileptiform abnormalities, remained within the range of variability observed between expert electroencephalographers.

These findings were confirmed in the large single‐center validation cohort of nearly 10,000 EEG recordings, where agreement between SCORE‐AI and routine clinical interpretations was again comparable to inter‐expert variability [36]. When benchmarked against previously published AI algorithms designed primarily for epileptiform discharge detection, SCORE‐AI maintained similar sensitivity while achieving substantially higher specificity, thereby improving overall diagnostic accuracy and reducing false‐positive classifications [36]. Unlike earlier approaches that focused on isolated EEG features, SCORE‐AI generates clinically meaningful diagnostic categories that more closely reflect routine EEG reporting and clinical workflows [36].

**FIGURE 1:**
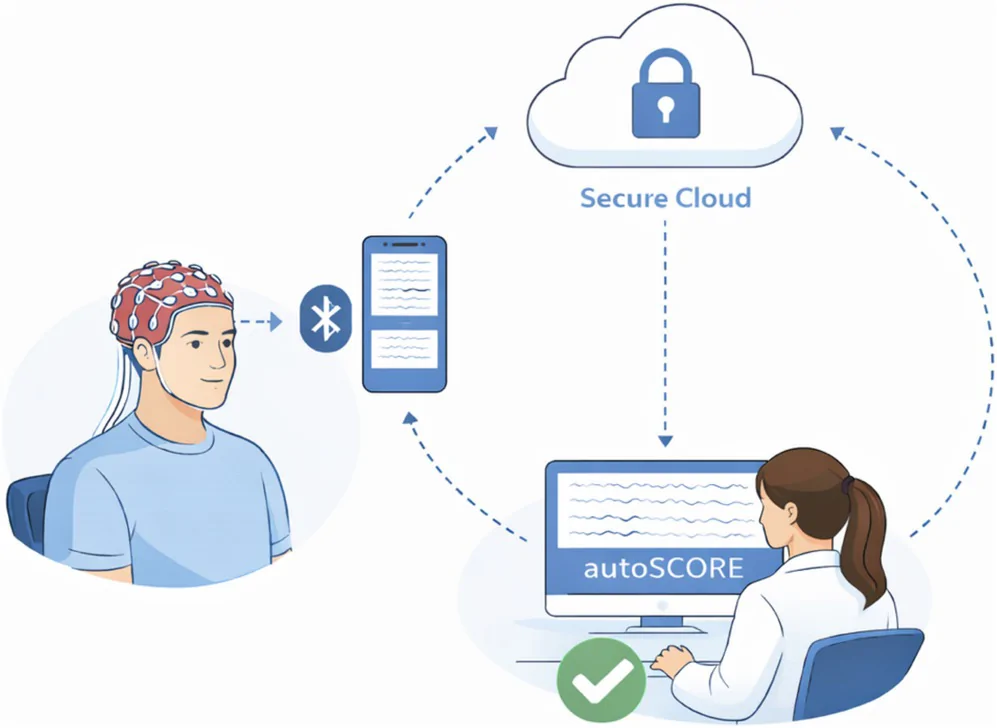
Cloud‐based semiautomated interpretation of EEGs, using low‐cost mobile EEGs and artificial intelligence.

The external validation study further demonstrated that the model generalized well across different ethnic populations and EEG acquisition systems [37]. When compared with an independent clinical reference standard, its diagnostic performance was equivalent to that of experienced EEG experts [37]. The model was subsequently implemented in a commercially available EEG reader (autoSCORE) and received regulatory approval both in the USA, the European Union, and in Australia. The possibility of deploying the system through cloud‐based platforms in combination with low‐cost mobile EEG devices (Figure 1) offers a promising strategy for expanding access to expert‐level EEG interpretation in resource‐limited settings where specialist expertise is scarce.

Taken together, these studies demonstrate that fully automated interpretation of routine EEGs with expert‐level performance is now achievable and has immediate potential to improve access to diagnostic services. Future developments should extend these approaches to additional patient populations, including neonates and critically ill patients, while integrating EEG interpretation with clinical outcomes and other diagnostic information. Prospective implementation studies, particularly in LMICs, will be essential to determine their real‐world clinical impact. Ultimately, such AI systems could support task‐sharing strategies, reduce diagnostic delays, and improve access to high‐quality epilepsy care at scale.

## 4 Automated Detection of GCS Using Wearable Devices

GCS are associated with substantial morbidity and an increased risk of sudden unexpected death in epilepsy (SUDEP) [38, 39]. Several medically certified wearable devices are currently available for automated GCS detection, including four commercial solutions, three approved by the U.S. Food and Drug Administration (Embrace/EpiMonitor, EpiWatch and NightWatch) and two carrying CE marking (EpiCare mobile/free and NightWatch) [40]. Despite their proven clinical utility, these technologies are largely confined to North America and Europe and remain virtually unavailable in LMICs. Their relatively high cost, which is often not reimbursed by healthcare systems, also represents a major barrier to widespread adoption, even in high‐income countries [40]. To improve accessibility, we developed EpiSave, a deep learning–based seizure detection system designed to operate on inexpensive, commercially available Android Wear OS smartwatches, with the objective of making the application freely available in LMICs [41].

EpiSave was developed using data from a prospective multicenter observational study conducted across eight European epilepsy monitoring units (EMUs). A total of 384 patients undergoing video‐EEG monitoring contributed more than 36,000 h of wrist accelerometer recordings, including 70 individuals who experienced at least one GCS during monitoring [41]. To ensure strict separation between model development and evaluation, the dataset was partitioned at the patient level into a training cohort comprising 37 patients with 54 GCS and an independent test cohort of 347 patients with 49 GCS.

The detection algorithm was based on a convolutional neural network (CNN) trained to analyze accelerometer amplitude signals derived from three‐dimensional wrist motion. To improve robustness and allow flexible optimization of detection performance, multiple neural networks were trained on different data partitions and combined using an ensemble strategy [41]. Individual predictions were aggregated through a quantile‐based approach, allowing sensitivity and false alarm rate (FAR) to be adjusted without retraining the underlying models. Performance was evaluated using clinically relevant event‐based metrics, including sensitivity, FAR, and detection latency, while extensive cross‐validation was performed to optimize model parameters. The final algorithm was subsequently implemented on a commercially available Wear OS smartwatch to verify its computational efficiency and feasibility for real‐time operation on widely accessible consumer devices.

The optimized model demonstrated excellent performance on the independent validation dataset, achieving a sensitivity of 96% (95% CI 90%–100%) with a FAR of approximately one false alarm every 8 days [41]. Performance remained consistent across patient subgroups, with most individuals experiencing no false alarms during the monitoring period. As expected, the ensemble architecture also enabled flexible adjustment of the operating point: sensitivity could be increased to nearly 100% at the expense of additional false alarms, whereas FAR could be reduced to approximately one false alarm every 100 days with only a modest decrease in sensitivity. This flexibility offers the possibility of tailoring detection thresholds to individual patient needs and clinical circumstances.

**FIGURE 2:**
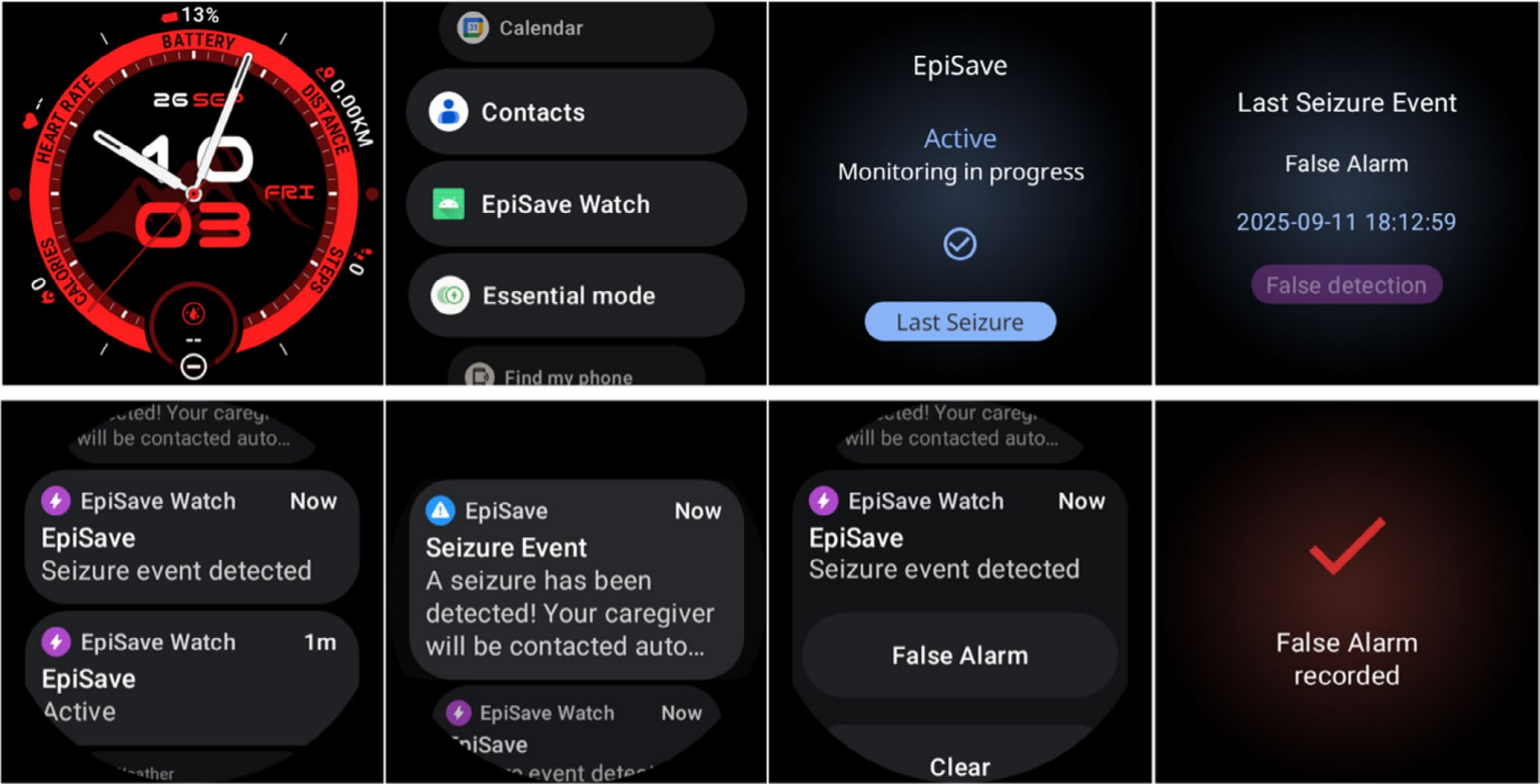
EpiSave app user interface as displayed on the screen of the smartwatch.

Detection was rapid, with a median latency of 26 s from seizure onset, and all detected seizures were identified before seizure termination [41]. This is particularly relevant from a clinical perspective because SUDEP‐related cardiorespiratory complications typically occur during the postictal period [38]. The algorithm also proved suitable for continuous real‐time execution on low‐cost smartwatches (Figure 2), with inference times well below the data acquisition interval. Notably, EpiSave relies exclusively on accelerometer signals, avoiding the need for additional physiological sensors and thereby facilitating implementation on affordable, widely available wearable devices [41].

These findings demonstrate that accurate detection of GCS can be achieved using a single wrist‐worn accelerometer integrated into commercially available low‐cost smartwatches, providing a realistic opportunity for large‐scale deployment. The next challenge will be to confirm performance under real‐world ambulatory conditions, where patient activity and environmental factors differ substantially from those encountered in epilepsy monitoring units. Longitudinal studies evaluating the impact of wearable seizure detection on clinical outcomes, including seizure‐related injuries and SUDEP prevention, will also be essential. For LMICs, where access to specialized epilepsy care remains limited, scalable and affordable wearable technologies such as EpiSave could represent an important step toward improving seizure monitoring and patient safety beyond specialized centers.

## 5 Discussion

The three examples presented in this review illustrate complementary applications of AI to address diagnostic and post‐diagnosis monitoring gaps in LMICs. First, a smartphone‐based diagnostic tool demonstrated that machine learning can support non‐specialist healthcare workers in identifying GCS with high accuracy in African settings. Second, automated EEG interpretation using deep learning achieved expert‐level performance, offering a scalable solution to the shortage of EEG expertise in LMICs [36]. Third, wearable‐based seizure detection showed that real‐time monitoring of GCS is feasible using low‐cost off‐the‐shelf devices [41]. Together, these approaches highlight the potential of AI to improve diagnosis, investigation, and monitoring of epilepsy in resource‐limited environments.

Nevertheless, the implementation of AI in epilepsy care in LMICs faces substantial challenges [3]. A primary limitation relates to infrastructure constraints, including unreliable electricity supply, limited internet connectivity, and insufficient access to digital devices [3, 42]. These factors can hinder the deployment, maintenance, and scalability of AI‐based solutions, particularly in rural or underserved regions. Even when mobile technologies are available, integration with existing healthcare systems, often still paper‐based, remains complex [43].

Another critical issue is the availability and quality of locally relevant data. Many AI models are trained on datasets derived from high‐income countries, raising concerns about generalizability to LMIC populations with different epidemiological profiles, comorbidities, and environmental exposures [44]. Bias in training data may lead to reduced accuracy and potential inequities in care [45]. Furthermore, the lack of standardized, large‐scale, and locally curated datasets limits the development of context‐adapted algorithms [46].

Human factors also represent an important barrier. Potentially limited digital literacy among primary healthcare workers, combined with high workload and resource constraints, may reduce adoption of AI tools [28]. In addition, there may be skepticism toward automated systems, particularly in settings where trust in healthcare systems is already fragile [47]. Importantly, AI tools should be viewed as augmenting, rather than replacing, clinical judgment, requiring appropriate training and governance frameworks. In addition, clear accountability frameworks are needed when AI‐supported recommendations prove to be incorrect. Defining the respective responsibilities of healthcare professionals, institutions, developers, and regulators will be essential to maintain patient safety and foster trust in AI‐assisted clinical decision‐making, particularly in healthcare systems where medico‐legal frameworks are still evolving [48].

Ethical and regulatory considerations are equally relevant. Issues related to data privacy, security, and informed consent are particularly sensitive in LMICs, where legal frameworks may be underdeveloped [42]. The risk of data misuse or breaches is amplified in environments with weak digital infrastructure [43]. Moreover, the deployment of AI without proper validation in local settings may lead to unintended consequences, including misdiagnosis, misuse or inappropriate management.

Finally, sociocultural factors, including stigma and health‐seeking behaviors, may influence the effectiveness of AI‐based interventions [13]. Without parallel efforts in education and community engagement, technological solutions alone are unlikely to fully address the epilepsy care gap [6].

Addressing these challenges requires a multifaceted and context‐sensitive approach. First, the co‐development with target users of low‐cost, resource‐efficient AI systems should be prioritized. Advances in edge computing and lightweight deep learning architectures allow algorithms to run locally on affordable devices, reducing dependence on internet connectivity and centralized infrastructure [49]. Integration of AI into portable and low‐cost EEG systems and widely available smartphones represents a particularly promising avenue for LMICs [36, 41].

Second, there is a critical need to generate locally relevant datasets and promote codesigning technologies with local communities. Collaborative initiatives involving local institutions, international organizations, and research networks could facilitate the collection of large, diverse datasets representative of LMIC populations [46]. Such efforts would improve model generalizability, reduce bias, and enable continuous algorithm refinement through iterative learning processes.

Third, task‐sharing strategies should be systematically integrated with AI deployment [12]. By empowering primary healthcare workers with AI‐supported diagnostic and monitoring tools, healthcare systems can extend the reach of specialized expertise. This approach aligns with global health priorities and has the potential to significantly reduce the epilepsy treatment gap, but also stigma. Such work must, though, be accompanied by appropriate training programs and clear clinical pathways to ensure safe and effective use.

Fourth, implementation research is essential [50]. Beyond technical validation, studies should evaluate the real‐world impact of AI tools on clinical outcomes, cost‐effectiveness, implementation strategies, and health system efficiency. Prospective trials in LMIC settings will be crucial to demonstrate adoption, scalability, and sustainability. Successful implementation will also depend on sustainable procurement, device maintenance, technical support, and integration into existing clinical workflows, all of which remain challenging in many LMICs. These operational aspects should be considered alongside diagnostic accuracy when evaluating AI‐based interventions [51].

In parallel, the development of ethical and regulatory frameworks tailored to LMIC contexts is needed. Initiatives such as trustworthy AI guidelines provide a foundation, but must be adapted to local realities, including considerations of equity, transparency, and accountability.

Finally, community engagement and education should not be overlooked [13]. Reducing stigma and improving awareness of epilepsy remain fundamental to improving care. AI‐based tools, when combined with targeted educational initiatives, may contribute to reshaping perceptions of epilepsy and promoting earlier access to treatment.

## 6 Conclusion

Artificial intelligence offers a unique opportunity to transform epilepsy care in LMICs by addressing critical gaps in diagnosis, investigation, and monitoring. While important challenges remain, the examples presented in this review demonstrate that scalable, low‐cost, and clinically meaningful solutions are already emerging. With continued collaboration between clinicians, researchers, and policymakers, AI‐driven approaches have the potential to substantially improve outcomes for people with epilepsy in resource‐limited settings and contribute to reducing the global epilepsy care gap.

## Author Contributions

**Jesper Tveit:** investigation, methodology, validation, writing – review and editing, software, formal analysis. **Harald Aurlien:** writing – review and editing, methodology, validation, formal analysis, software, investigation. **Adriano Bernini:** writing – review and editing, project administration, investigation. **Gabriel Davis Jones:** conceptualization, investigation, methodology, validation, formal analysis, software, writing – review and editing. **Symon M. Kariuki:** investigation, validation, writing – review and editing. **Arjune Sen:** conceptualization, supervision, investigation, writing – review and editing, funding acquisition. **Antoine Spahr:** writing – review and editing, software, formal analysis, methodology, validation. **Sándor Beniczky:** conceptualization, funding acquisition, writing – review and editing, supervision, data curation, methodology, validation, formal analysis, investigation. **Philippe Ryvlin:** conceptualization, writing – original draft, supervision, data curation, funding acquisition, validation, investigation.

## Funding

This work was supported by Schweizerischer Nationalfonds zur Förderung der Wissenschaftlichen Forschung, #320030_179240, #CRSII5_193813, #3200‐0‐242953. National Institute for Health and Care Research, NIHR304306. HORIZON EUROPE Research Infrastructures, 101147319.

## Conflicts of Interest

The authors declare no conflicts of interest. Harald Aurlien is Senior Director, Clinical Research, Natus Medical, Bergen, Norway. Jesper Tveit is Senior BI Developer, Natus Medical, Bergen Norway.

## Data Availability

Data sharing not applicable to this article as no datasets were generated or analysed during the current study.
